# Humoral Responses to Repetitive Doses of COVID-19 mRNA Vaccines in Patients with CAR-T-Cell Therapy

**DOI:** 10.3390/cancers14143527

**Published:** 2022-07-20

**Authors:** Simona Gössi, Ulrike Bacher, Claudia Haslebacher, Michael Nagler, Franziska Suter, Cornelia Staehelin, Urban Novak, Thomas Pabst

**Affiliations:** 1 Department of Medical Oncology, Inselspital, Bern University Hospital, 3010 Bern, Switzerland; simona.goessi@students.unibe.ch (S.G.); claudia.haslebacher@insel.ch (C.H.); urban.novak@insel.ch (U.N.); 2 Department of Hematology, Inselspital, Bern University Hospital, 3010 Bern, Switzerland; veraulrike.bacher@insel.ch; 3 University Institute of Clinical Chemistry (UKC), Inselspital, Bern University Hospital, 3010 Bern, Switzerland; michael.nagler@insel.ch; 4 Institute for Infectious Diseases (IFIK), Inselspital, University of Bern, 3010 Bern, Switzerland; franziska.suter@ifik.unibe.ch; 5 Department of Infectiology, Inselspital, University of Bern, 3010 Bern, Switzerland; cornelia.staehelin@insel.ch

**Keywords:** CAR-T-cell therapy, mRNA COVID-19 vaccines, humoral antibody responses, diffuse large B-cell lymphoma (DLBCL)

## Abstract

**Simple Summary:**

Data on the efficacy of SARS-CoV-2 mRNA vaccinations in patients with CAR-T-cell therapy is very limited. We analyzed patients (predominantly DLBCL) undergoing CAR-T-cell therapy and receiving BNT162b2 (Pfizer-BioNTech) or mRNA-1273 (Moderna) vaccination. This single center retrospective analysis aimed to evaluate the number of B-cells and CAR-T-cell copies as prognostic factors of humoral antibody test results as well as the effects of a third and fourth dose on humoral antibody response. Our results demonstrate that patients with more B-cells and fewer CAR-T-cells at vaccination were more likely to produce a positive antibody test result. Overall, we found very poor humoral antibody responses, while additional doses increased rates of seroconversion and antibody titers.

**Abstract:**

*Background*: Due to B-cell aplasia following CAR-T-cell therapy, patients are at risk of severe SARS-CoV-2 course. *Methods*: COVID-19 vaccines were assessed by IgG antibody tests against SARS-CoV-2 spike protein (anti-S1/S2). Vaccination procedures: group (1): CAR-T-cells followed by two to four vaccine doses; group (2): Two vaccine doses prior to CAR-T-cells, followed by doses 3 or 4. *Results*: In group 1 (*n* = 32), 7/30 patients (23.2%) had positive antibody tests after a second dose, 9/23 (39.1%) after a third dose, and 3/3 patients after a fourth dose. A third dose led to seroconversion in 5 of 21 patients (23.8%) with available data, while a fourth dose did so in 2/3 patients. Higher B-cells (AUC: 96.2%, CI: 89–100, *p* = 0.0006) and lower CAR-T-cell copies (AUC: 77.3%, CI: 57–97, *p* = 0.0438) were predictive of positive humoral vaccine response. In group 2 (*n* = 14), 6/14 patients (42.9%) had a positive antibody test after a second dose, 3/8 patients (37.5%) after a third dose, and 3/4 patients after a fourth dose. A third dose led to seroconversion in 1/8 patients (12.5%), while a fourth dose did so in 3/4 patients. *Conclusion*: Additional vaccine doses increased seroconversion rates whilst high B-cell counts and low CAR-T-cell copy numbers were associated with positive antibody response.

## 1. Introduction

CAR-T-cell therapy is a highly promising therapeutic option in the treatment of advanced lymphoproliferative neoplasms such as diffuse large B-cell lymphoma (DLBCL), acute lymphatic leukemia (ALL), and mantle cell lymphoma [1,2,3,4]. CD19-directed CAR-T-cells have an impact on malignant B-cell tissues as well as the healthy B-cell compartment and thus lead to B-cell depletion and hypogammaglobulinemia [5,6]. Frequent complications are Cytokine release syndrome (CRS) in more than 60% and CAR-T-Related Encephalopathy Syndrome (CRES) in more than 30% of the patients, according to the literature [7]. Increased serum IL-6 levels and clinical CRS symptoms can contribute to the indication of therapeutic interventions [8]. Tocilizumab should be administered to patients with CRS, while corticosteroids are used in patients with CRES and CRS not responsive to tocilizumab [6].

Patients with CAR-T-cell therapy suffer from severe immunosuppression and thus are particularly vulnerable to infectious diseases, such as COVID-19. This is due to the prolonged cytopenias with B-cell aplasia and hypogammaglobulinemia caused by CAR-T-cell therapy. Also, treatment of CAR-T-cell complications with tocilizumab and steroids has been shown to further increase these patients’ vulnerability [9]. Spanjaart et al. have found that patients diagnosed with a COVID-19 infection after B-cell targeted CAR-T-cell therapy have a COVID-19 attributable mortality rate of 41%. Therefore, in the context of the COVID-19 pandemic, it is crucial to protect the patients undergoing CAR-T-cell therapy with an effective vaccination against COVID-19. The mRNA-COVID-19 vaccines of Pfizer/BioNTech Comirnaty (BNT162b2) and Moderna (mRNA-1273) were approved by the European Medicines Agency and are widely used [10]. The European Society for Blood and Marrow Transplantation (EBMT) recommends maintaining a period of 6 months between CAR-T-cell therapy and vaccination, due to delayed B-cell reconstitution. After this interval, three doses are recommended as a primary series, at 4 week intervals [11]. However, data on the efficacy of the vaccinations for CAR-T-cell patients are sparse.

Dhakal et al. have reported a seropositivity rate of only 21% (3/14 patients) after two doses of the mRNA vaccine in patients with CAR-T-cell therapy [12]. Similarly low percentages have been reported by Ram et al. with a positive serology of 36% (5/14 patients) in patients after CAR-T-cell therapy and two doses of the BNT162b2 mRNA COVID-19 vaccine [13]. However, the efficacy of a third dose in patients with CAR-T-cell therapy has yet to be examined. A small study identified patients who had no humoral response after two vaccine doses and eventually achieved a positive serology after a third vaccine dose in 40% (4/10 patients) of patients post allogeneic HCT and in 17% (1/6 patients) post CAR-T-cell therapy [14]. Currently, there is no data on the efficacy of a fourth dose of a COVID-19 vaccine in patients with CAR-T-cell therapy. Additionally, patients vaccinated before CAR-T-cell therapy face another problem, as the therapy procedures often wipe out all immune memory of other vaccines. It is therefore recommended by the EBMT to re-vaccinate these individuals as if they had never received a COVID-19 vaccine [11]. This recommendation is based on evidence from other vaccines, where revaccination after CAR-T-cell therapy is also recommended [15].

There are still many open questions regarding optimal timing of vaccination after CAR-T-cell therapy as well as the efficacy of different amounts of vaccine doses. In addition, re-vaccination strategies of patients with vaccination prior to CAR-T-cell therapy have yet to be clarified. This analysis attempted to provide further details to this limited data, analyzing humoral antibody responses to COVID-19 vaccines in CAR-T-cell therapy patients. In this real-world retrospective approach, we studied two consecutive cohorts: (A) patients who received two to four doses of the vaccine after CAR-T-cell therapy since vaccination was simply not yet available at CAR-T treatment, and (B) patients with two doses before CAR-T-cell therapy and subsequent re-vaccination after CAR-T treatment. Finally, the impacts of B-cell and CAR-T-cell levels in patients’ peripheral blood were assessed at the time of the first vaccination as well as the relevance of the interval between CAR-T-cell infusion and subsequent vaccination.

## 2. Materials and Methods

### 2.1. Methods

We conducted a retrospective single-center study at the University Hospital Inselspital, Bern, Switzerland. Patients (predominantly DLBCL, mantle cell lymphoma, B-ALL) undergoing CAR-T-cell therapy between January 2019 and December 2021 were analyzed. They received two to four doses of COVID-19 mRNA vaccines between January 2021 and February 2022. We assessed the COVID-19 vaccines using IgG antibodies against SARS-CoV-2 spike protein (anti-S1/S2) (Clia Diasorin; cut-off > 12 AU/mL for minimal positive results and cut-off > 100 AU/mL for clear positive results). IgG antibody test results were compared after the second, third, and fourth dose of the mRNA COVID-19 vaccine. In patients with CAR-T-cell therapy before vaccination, the number of B-cells and CAR-T-cells in peripheral blood at the time of first vaccination and the interval between CAR-T-cell infusion and first vaccination were analyzed. Additionally, we analyzed the effect of age at the time of vaccination on the antibody response to control for any bias due to the substantial age range in our cohort. The purpose of this analysis was to assess the possible influence of these parameters related to CAR-T-cell therapy before vaccination on the results of the IgG antibody test. The infection rate of the patients is based on medical history and anamnesis. Data on eventual COVID-19 PCR test results were not available for this analysis.

### 2.2. Patients

**Figure 1:**
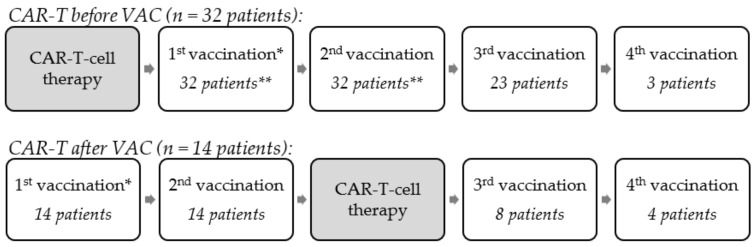
Illustration of vaccination procedures. * No antibody determination after the 1st vaccination. ** In the group “CAR-T before VAC”, 32 patients had a 1st and 2nd vaccination, but data on antibody determination after 2nd vaccination were only available for 30 patients.

Patients received vaccinations depending on availability of the vaccines throughout the evolution of the pandemic, as well as the treating decisions of the attending physicians. Retrospectively, we analyzed the vaccination procedure. To this end, we divided the patients retrospectively into two groups depending on their vaccination status prior to CAR-T-cell therapy. This allowed a more differentiated analysis of the data in these two different vaccination settings. In group 1, which will be called “CAR-T before VAC”, vaccines were not yet available before the CAR-T-cell therapy. The procedure in these patients was as follows: Administration of CAR-T-cell therapy followed by either two, three, or four doses of the COVID-19 vaccine. In group 2, which will be called “CAR-T after VAC”, the procedure was as follows: Administration of two doses of COVID-19 vaccines, followed by CAR-T-cell therapy, and then re-vaccination with doses 3 or 4 of the COVID-19 vaccine. Figure 1 is an illustration of these vaccination procedures. Informed consent was obtained from all subjects involved in the study.

### 2.3. Laboratory Analysis

#### 2.3.1. Antibody Determination

The quantitative analysis of anti-S1 and anti-S2 IgG against SARS-CoV-2 in human serum was based on an indirect chemiluminescence immuno assay (CLIA).

In the first step, human serum is incubated with recombinant S1- and S2-coated magnetic beads. Anti-SARS-CoV-2 IgG antibodies present in human serum bind to S1 and S2 antigens. Unbound antibodies are removed by washing cycles. In the second step, isoluminol-conjugated mouse anti-human antibodies are added to bind immobilized anti-SARS-CoV-2 IgG captured in the solid phase. Unbound isoluminol-conjugated antibodies are removed by washing cycles. In the last step, starter reagents are added to evoke a chemiluminescence light reaction. Luminescence is measured in relative light units (RLU) and converted to antibody concentrations in arbitrary units per milliliter (AU/mL) by the automated immunoassay analyzer Liaison^®^ XL by DiaSorin, Saluggia, Italy. CLIA is very similar to an ELISA. The difference is that in an ELISA, the readout is based on a colorimetric reaction and subsequent quantification by absorption, whereas in a CLIA, the readout is based on measuring luminescence signal. Sensitivity of the assay was as follows: ≤5 days: 25% (14.6–39.4%); 5–15 days: 90.4% (79.4–95.8%); and >15 days: 97.4% (86.8–99.5%). The specificity of the assay was 98.9 % (94.0–99.8 %). Of note, in this assay, it is not possible to distinguish between anti-S1 and anti-S2 IgG. RBD is part of the S1 subunit of the SARS-CoV-2 spike protein; therefore, binding to RBD is also considered. Meanwhile, there are tests commercially available specifically targeting RBD alone or the whole trimer of the spike protein.

#### 2.3.2. B-Cells

B-cells were measured by multiparameter flow cytometry using the TBNK Multitest kit (including CD4/CD8, CD19, CD16+/CD56+) on the Lyrics platform (kit and platform, both from BD Biosciences, Allschwil, Switzerland). For absolute cell count measurement, this system includes tubes with a defined number of fluorescent Latex beads (“TruCOUNT”). Alternatively, for some samples, we used the BD OneFlowTM Lymphoid Screening Tube (LST), which includes mature lymphocyte populations of B, T, and NK lineages (including the B-cell antigens CD19, CD20, skappa/slambda) (previously Cytognos, now also BD Biosciences, Allschwil, Switzerland). SARS-CoV-2 specific memory B-cells had not been determined in these patients, as B-cells were only analyzed before vaccination.

### 2.4. Statistical Analyses

For the statistical analyses, we used GraphPad Prism and we performed One-Way ANOVA in a mixed effect model with a follow-up Tukey test for the comparison of the IgG antibody test results. For the analysis of prognostic parameters, we used simple logistic regression.

## 3. Results

### 3.1. Clinical Characteristics of the Patients

The clinical characteristics of the 46 patients are presented in Table 1. Median age of patients at time of diagnosis was 58.5 years, and the male/female ratio was 1.6. The initial diagnosis was de novo DLBCL (52%; 24/46), transformed DLBCL (24%; 11/46), mantle cell lymphoma (15%; 7/46), and B-ALL (9%; 4/46). Previous autologous stem cell transplantation before CAR-T-cell therapy had been conducted in 21 patients (46%) and allogeneic stem cell transplantation before CAR-T-cell therapy in 1 patient (2%). The median age at the time of CAR-T-cell therapy was 64 years. A total of 25 patients had the CAR-T-cell therapy tisagenlecleucel (61%; 28/46), 10 patients had axicabtagene ciloleucel (22%; 10/46), and 8 patients had brexucabtagen autoleucel (17%; 8/46). A total of 30 patients suffered from a Cytokine release syndrome (grade I–III) (65%; 30/46), while 12 patients suffered from a CAR-T-Related Encephalopathy Syndrome (grade I-IV) (26%; 12/46). A total of 21 patients were treated with Tocilizumab (46%; 21/46), and 16 with steroids (35%; 16/46). A total of 10 patients had a relapse after CAR-T-cell therapy (22%; 10/46), and 3 patients died during the follow-up of this study (7%; 3/46).

### 3.2. Vaccination Procedures

Retrospectively, we assessed the effectivity of the vaccinations of the CAR-T-cell patients. Overall, we identified two vaccination procedures. The retrospective distribution of patients into two groups is illustrated in Figure 1. We observed 32 patients with their CAR-T-cell therapy before a COVID-19 vaccination (“CAR-T before VAC”). In total, 32 patients were identified with two vaccinations after the CAR-T-cell therapy (but only 30 patients with an antibody test result after second vaccination, as in two patients, data was not available due to the retrospective data collection). In total, 23 of these patients also received a third vaccination and 3 patients a fourth vaccination. Finally, we identified 14 patients with their CAR-T-cell therapy after a COVID-19 vaccination (“CAR-T after VAC”). In total, 14 patients were identified with two vaccinations before the CAR-T-cell therapy; we found that 8 of these patients received a third dose after the CAR-T-cell therapy, and 4 patients received a fourth dose.

### 3.3. Parameters Concerning Vaccination

Table 2 shows all the parameters concerning vaccination. The first vaccine in 33 patients (72%; 33/46) was Moderna (mRNA-1273), while 13 patients (28%; 13/46) were administered the vaccine of Pfizer/BioNTech (BNT162b2). All patients, except for one, had the same vaccine for all following doses. Only one patient had the first two doses with Moderna and received the third booster dose with Pfizer/BioNTech. The remission status at the time of first vaccination was complete remission (46%; 21/46), partial remission (11%; 5/46), progressive disease (28%; 13/46), and not evaluated (15%; 7/46).

In total, 32 patients with the “CAR-T before VAC” procedure were identified. The median time between CAR-T-cell therapy and the first vaccination was 286 days, while the median time between CAR-T-cell therapy and second vaccination was 317 days. The median time between second vaccination and antibody determination was 57 days.

In total, 14 patients with the “CAR-T after VAC” vaccination procedure were identified. The first vaccination had a median of 76 days and the second vaccination had a median of 37 days before CAR-T-cell therapy. The median time between second vaccination and antibody determination was 40 days. The third vaccination took place a median of 102 days after CAR-T-cell therapy.

### 3.4. Humoral Antibody Responses

#### 3.4.1. CAR-T before VAC (*n* = 32 Patients)

The humoral antibody titers after second, third, and fourth vaccination in patients with CAR-T-cell therapy before vaccination are illustrated in Table 3. In patients with the procedure “CAR-T before VAC”, we identified 32 patients who received at least two doses of the COVID-19 vaccination after their CAR-T-cell therapy. Two patients received no antibody determination after two vaccine doses. Negative antibodies were found in 23 patients (76.7%; 23/30), while 7 patients (23.3%; 7/30) had positive humoral antibody responses, among whom 5 patients (16.6%; 5/30) had clear positive antibody titers > 100 AU/mL.

Furthermore, in 23 patients, data were available after a third dose of the COVID-19 vaccination after their CAR-T-cell therapy. Negative antibodies were found in 14 patients (60.8%; 14/23), while 9 patients (39.1%; 9/23) had positive humoral antibody responses (only 5 patients (21.7%; 5/23) with clear positive antibody titers > 100 AU/mL). In 21 of these patients, data were available after both the second and third dose of the vaccination. In total, 10 patients (47.6%; 10/21) stayed negative after the second and third dose, while in 5 patients (23.8%; 5/21), a negative result after the second vaccine could be turned into a positive result after the third dose (2 patients (9.5%; 2/21) with a clear positive result after third vaccination). Six patients (28.6%; 6/21) already had a positive test result after two doses, which increased due to third vaccination in four patients (19%; 4/21).

Finally, in three patients, data were available after a fourth vaccination after their CAR-T-cell therapy. All three patients evaluated with four vaccine doses had positive humoral antibody responses (only 1pt. (1/3) with clear positive antibody titers > 100 AU/mL). Two patients seroconverted after the fourth vaccination, while 1pt. stayed clear positive after the third and fourth vaccination.

#### 3.4.2. CAR-T after VAC (14 Patients)

The humoral antibody titers after second, third, and fourth vaccination in patients with CAR-T-cell therapy after vaccination are illustrated in Table 4. In patients with the procedure “CAR-T after VAC”, there were 14 patients who received at least two doses of the COVID-19 vaccination before their CAR-T-cell therapy. Among these, eight patients had negative humoral antibody responses (57.1%; 8/14), while six patients (42.9%; 6/14) had positive humoral antibodies (only 1pt. (7.1%; 1/14) with clear positive antibody titers > 100 AU/mL).

Of these patients, eight received a third dose of the COVID-19 vaccination after their CAR-T-cell therapy. Negative antibodies were found in five patients (62.5%; 5/8), while three patients (37.5%; 3/8) had positive humoral antibody responses (only 1pt. (12.5%; 1/8) with clear positive antibody titers > 100 AU/mL). In all of these eight patients, data were available after the second and third doses of the vaccination. Four patients (50%; 4/8) remained negative after the second and third dose, while 1 pt. (12.5%; 1/8) seroconverted after the third dose. Three patients (37.5%; 3/8) already had a positive test result after two doses, and this increased due to a third vaccination in two patients (25%; 2/8).

Finally, four patients received a fourth dose of the vaccination after their CAR-T-cell therapy. Negative antibodies were found in one patient (1/4), while three patients (3/4) had positive humoral antibody responses (only two patients (2/4) with clear positive antibody titers > 100 AU/mL). In three patients (3/4), we noted seroconversion after the fourth vaccination (two patients (2/4) with clear positive results after the fourth vaccination), while one patient (1/4) stayed negative after the third and fourth vaccinations.

### 3.5. Anti-Spike Protein IgG Antibody Titers

Due to our small sample size, we found distinct differences in the point estimates (mean differences of titer levels between specific vaccinations), but none of these showed statistical significance.

#### 3.5.1. CAR-T before VAC (32 Patients)

In patients with “CAR-T before VAC”, the third dose of the COVID-19 vaccination in comparison with the second dose led to an increase from 34.65 AU/mL to 74.8 AU/mL (mean diff. 40.15 AU/mL; SE of diff. 19.33; *p* = 0.1202). The fourth dose in comparison to the third dose led to an increase from 74.8 AU/mL to 116.1 AU/mL (mean diff. 41.3 AU/mL; SE of diff. 44.72 AU/mL; *p* = 0.6804). Comparing the second to the fourth dose of the vaccination, there was an increase from 34.65 AU/mL to 116.1 AU/mL (mean diff. 81.48; SE of diff. 34.6 AU/mL; *p* = 0.2475).

**Figure 2:**
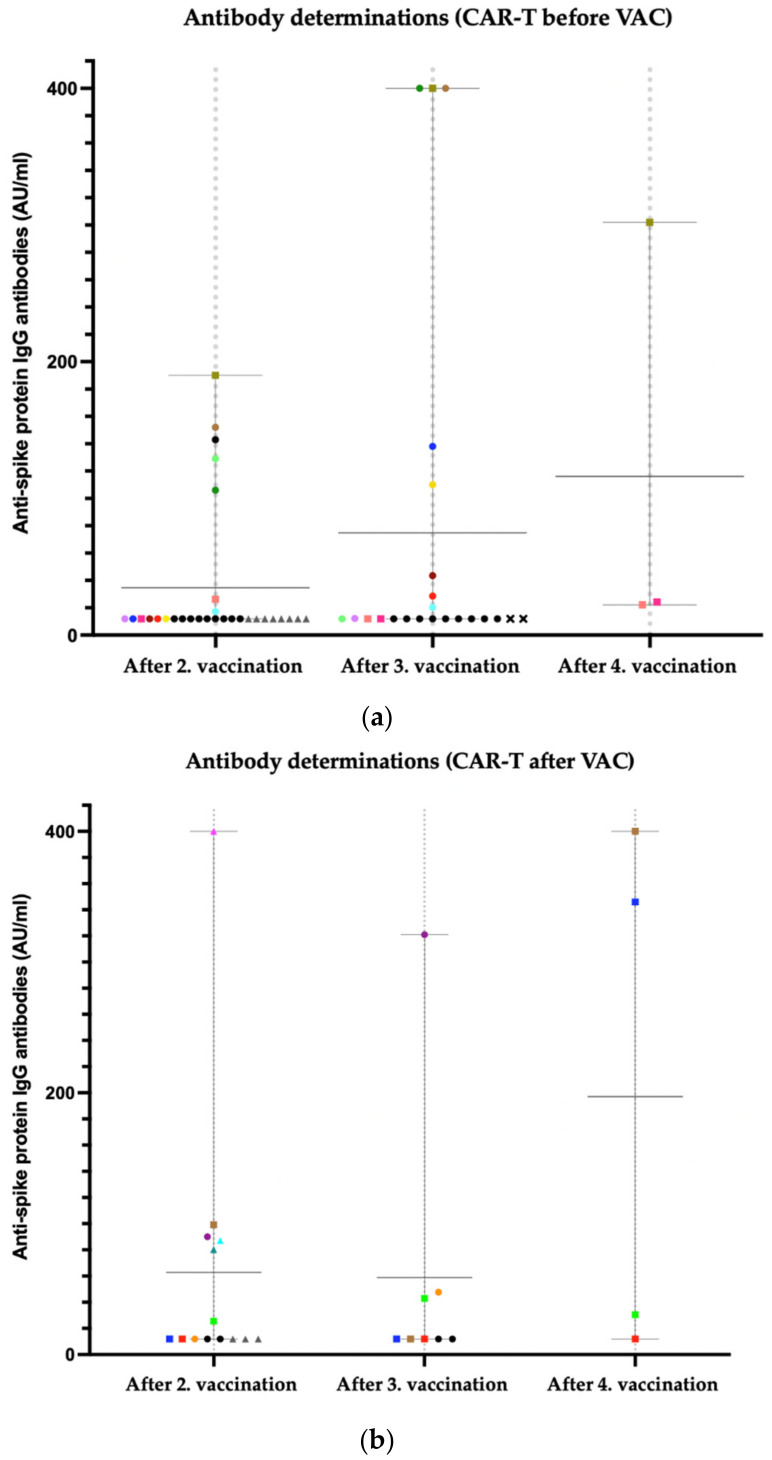
(**a**) Anti-spike protein IgG antibody titers after 2nd, 3rd, and 4th vaccination in patients with “CAR-T before VAC”; Square: In this patient, data after 2nd, 3rd, and 4th vaccination are available; Circle: in this patient, data after 2nd and 3rd vaccination are available; Triangle: in this patient, data after 2nd vaccination are available; X: in this patient, data only after third vaccination are available; eight patients had an antibody titer of <12AU/mL after 2nd vaccination (grey triangle) and nine patients had an antibody titer of <12AU/mL after 2nd and 3rd vaccination (black circle). (**b**) Anti-spike protein IgG antibody titers after 2nd, 3rd, and 4th vaccination in patients with “CAR-T after VAC”. Square: in this patient, data after 2nd, 3rd, and 4th vaccination are available; Circle: in this patient, data after 2nd and 3rd vaccination are available; Triangle: in this patient data, after 2nd vaccination are available; three patients had an antibody titer of <12AU/mL after 2nd vaccination (grey triangle) and two patients had an antibody titer of <12AU/mL after 2nd and 3rd vaccination (black circle).

In Figure 2a, we illustrate IgG antibody titers after second (*n* = 30 patients), third (*n* = 23 patients), and fourth (*n* = 3 patients) vaccination in patients with CAR-T-cell therapy before vaccination. Each point in the columns shows data of one individual patient. We also illustrate the mean values with range after second (mean 34.65 AU/mL; range 12–190), third (mean 74.8 AU/mL; range 12–400), and fourth vaccination (mean 116.1 AU/mL; range 22.1–302). Please note that all patients with an antibody determination of <12 AU/mL are depicted at 12 AU/mL in this graph.

#### 3.5.2. CAR-T after VAC (*n* = 14 Patients)

In patients with “CAR-T after VAC”, the third dose of the COVID-19 vaccination in comparison with the second dose led to a slight decrease from 62.71 AU/mL to 58.94 AU/mL (mean diff. −3.77 AU/mL; SE of diff. 30 AU/mL; *p* = 0.9913). The fourth dose in comparison to the third dose led to an increase from 58.94 AU/mL to 197.1 AU/mL (mean diff. 138.2AU/mL; SE of diff. 94.39 AU/mL; *p* = 0.4196). Comparing the second to the fourth dose of the vaccination, there was an increase from 62.71 to 197.1 AU/mL (mean diff. 134.4 AU/mL; SE of diff 74.05AU/mL; *p* = 0.3055).

In Figure 2b, we illustrate IgG antibody titers after second (*n* = 14 patients), third (*n* = 8 patients), and fourth (*n* = 4 patients) vaccination in patients with CAR-T-cell therapy after vaccination. Each point in the columns shows data of one individual patient. The figure also illustrates the mean antibody levels with the corresponding ranges after second (mean 62.71 AU/mL; range 12–400), third (mean 58.94 AU/mL; range 12–321), and fourth vaccination (mean 197.1 AU/mL; range 12–400). Please note that all patients with an antibody level <12 AU/mL are depicted at 12 AU/mL in this graph.

### 3.6. Prognostic Factors on the Antibody Outcome (CAR-T before VAC; n = 32 Patients)

In this study, we examined the number of B-cells and CAR-T-cells at time of vaccination, the time interval between CAR-T-cell therapy and first vaccination, and the age at time of vaccination as prognostic factors on the antibody outcome.

In 28 of the 32 identified patients who received a vaccination after CAR-T-cell therapy, data regarding the amount of B-cells in the blood of the patients were available. This blood sample of B-cells was taken a median of 34 days before the first vaccination. All 21 patients with 0/uL B-cells in this blood sample at the time of the first vaccination had a negative antibody test result after the second vaccination. The more B-cells (optimal cutoff at >10/uL B-cells; PPV 85.7%; NPV 100%) in the patients’ peripheral blood at the time of the first vaccination, the more likely patients were to develop a positive antibody test result after the second vaccination (area under the curve (AUC): 96.2%, CI: 89–100, *p* = 0.0006).

In 28 of the 32 identified patients who received a vaccination after CAR-T-cell therapy, data regarding the amount of CAR-T copies in the blood of the patients were available. This blood sample of CAR-T copies was taken a median of 30 days before the first vaccination. The fewer the CAR-T copies (optimal cutoff at <38/µg DNA CAR-T copies; PPV 94.7%; NPV 55.5%) in the patients’ peripheral blood at the time of the first vaccination, the more likely patients were to develop a positive antibody test result after the second vaccination (area under the curve (AUC): 77.3%, CI: 57–97, *p* = 0.0438).

**Figure 3:**
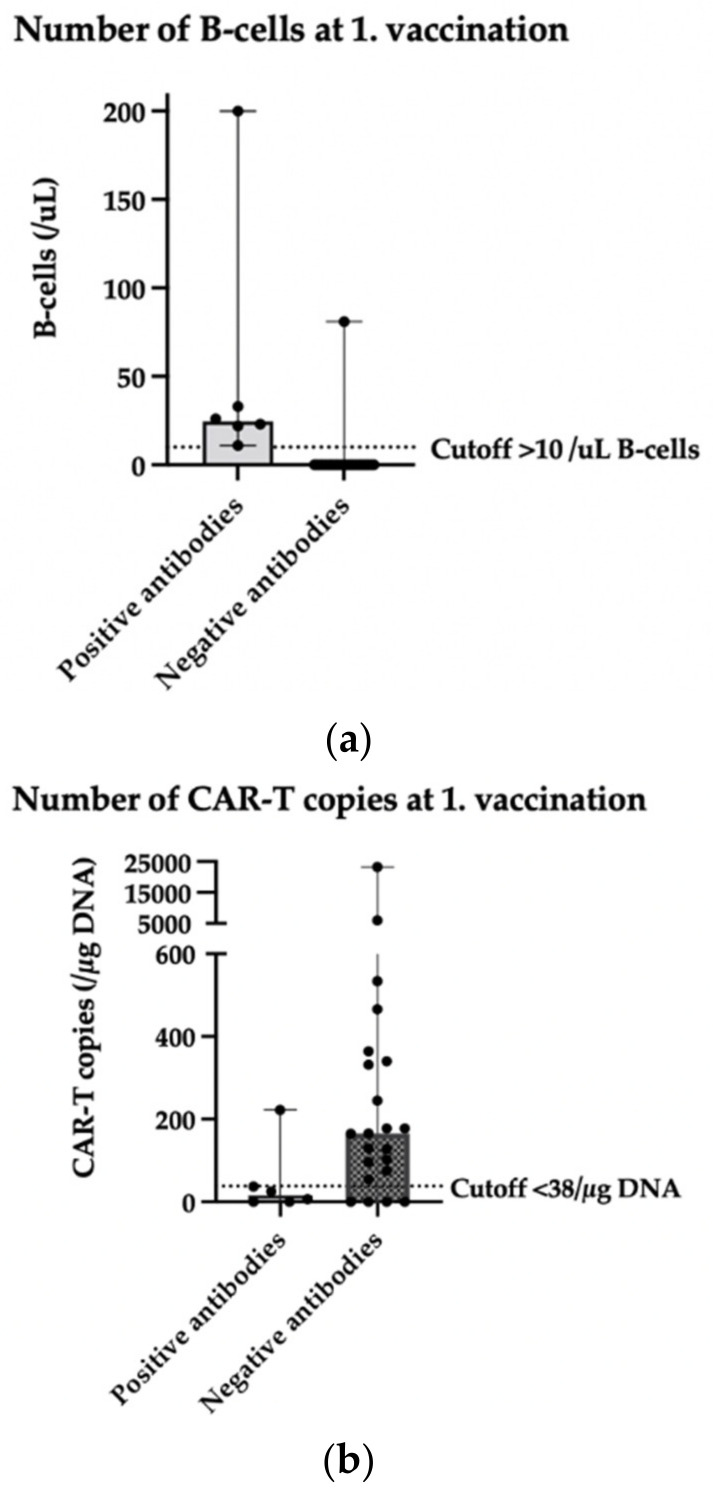
Scatter plot (median with range) depicting (**a**) number of B-cells at time of 1st vaccination and (**b**) number of CAR-T-cell copies at time of 1st vaccination in patients with positive anti-spike protein IgG antibody test result (>12AU/mL) compared to patients with a negative antibody test result. (**a**) *n* = 28 patients (21 patients with negative antibodies overlap at 0/uL B-cells); (**b**) *n* = 28 patients.

The influence of B-cells and CAR-T-cells at first vaccination on antibody outcome is shown in Figure 3. The distribution of B-cells and CAR-T-cell copies in patients with positive and negative antibodies is illustrated.

The time interval between CAR-T-cell therapy and first vaccination was tested as a prognostic parameter for the antibody outcome after the second vaccination. In 30 of the 32 patients with vaccination after CAR-T-cell therapy (2 patients had no antibody evaluation after second vaccination), we found no significant correlation between time interval and antibody outcome (AUC: 65.8%, CI: 41–89, *p* = 0.2112). The time interval between CAR-T-cell therapy and vaccination was ≤6 months in 8 patients, while it was >6 months in 22 patients. Overall, 1/8 patients with a time interval of ≤6 months had a positive antibody response, whereas 6/22 patients with a time interval of >6 months had a positive antibody response. These differences are not statistically significant.

Due to the substantial age range in patients, we also tested the age at time of first vaccination as a prognostic parameter for the antibody outcome after the second vaccination. In 30 of the 32 patients with vaccination after CAR-T-cell therapy (2 patients had no antibody evaluation after 2nd vaccination), we found no significant correlation between age at time of first vaccination and antibody outcome (AUC: 50.6%, CI: 27–73, *p* = 0.9609). Overall, 5 patients were under the age of 50 years, while 25 patients were more than 50 years old. Only 1/5 patients < 50 years in age had a positive antibody response, whereas 6/25 patients > 50 years of age had a positive antibody test result. These differences are not significant.

### 3.7. Infection Rate

Overall, 12 out of 46 patients reported a COVID-19 infection in their medical history. Eight patients had no detectable antibody response before the infection. Four patients had a positive antibody response before the infection (but only 1/4 with a strong positive result > 100 AU/mL). In seven patients, the infection took place after the second vaccination, in four patients after the third vaccination, and in one patient after the fourth vaccination.

Taking a closer look at the groups, 9/32 patients with CAR-T before VAC were infected. Four patients with reported infection were infected after second vaccination. These were 4/23 patients with negative antibodies and 0/7 patients with positive antibodies. Four patients were infected after third vaccination. These were 3/14 patients with negative antibodies and 1/9 patients with positive antibodies. One patient was infected after fourth vaccination. These were 1/3 patients with positive antibodies.

Overall, 3/14 patients with CAR-T after VAC were infected. All three patients with reported infection were infected after second vaccination. These were 1/8 patients with negative antibodies and 2/6 patients with positive antibodies (only 1 infected patient with a strong positive result > 100AU/mL) after second vaccination.

## 4. Discussion

In this retrospective real-world analysis, performed at a single tertiary academic center, we examined patients with CAR-T-cell therapy and two to four doses of COVID-19 vaccination and two different vaccination procedures. 

In patients who underwent CAR-T-cell therapy before COVID-19 vaccination, we found a very low seropositivity rate of only seven patients (23.3%; 7/30) after two doses of the vaccine. Other studies have shown similar low results, with a seropositivity rate of 21% (3/14 patients) in the study from Dhakal et al. and 36% (5/14) in the study from Ram et. al. [12,13]. Administration of a third dose lead to seroconversion in five patients (23.8%; 5/21) and an increase of antibody titers in four patients (19%; 4/21). Ram et al. showed similar results with post CAR-T-cell patients who had no humoral response after two doses, in which 17% (1/6) of patients achieved a positive result after a third dose [14]. Our study showed that seroconversion could be achieved in two patients (66%; 2/3) due to the fourth vaccination, while 1pt. (33%; 1/3) stayed clearly positive after third and fourth vaccination. Currently, there is very limited data on a fourth dose in immunosuppressed patients, but a study from Alejo et al. with 18 solid organ transplant recipients showed that 50% of participants with negative and all with low-positive titers showed significant boosting to high-positive titers after the fourth dose [16]. Therefore, our study suggests a clear benefit from a third as well as a fourth vaccination in patients with prior CAR-T-cell therapy, as it could lead to an increase in the number of patients with a positive antibody result and an increase in anti-spike protein IgG antibody titers.

In this analysis, the number of B-cells and CAR-T copies in patients’ peripheral blood at time of first vaccination and the time interval between CAR-T-cell therapy and first vaccination were investigated as a prognostic factor for the outcome of antibody tests in patients with prior CAR-T-cell therapy. We found that all 21 patients with 0/µL B-cells at the time of the first vaccination had a negative antibody test result. The more B-cells (optimal cutoff at >10/µL B-cells; PPV 85.7%; NPV 100%) in the patients’ peripheral blood at the time of the first vaccination, the more likely patients were to develop a positive antibody test result (AUC: 96.2%, CI: 89–100, *p* = 0.0006). Also, the fewer the CAR-T copies (optimal cutoff at <38/µg DNA CAR-T copies; PPV 94.7%; NPV 55.5%) in the patients’ peripheral blood at the time of the first vaccination, the more likely patients were to develop a positive antibody test result (AUC: 77.3%, CI: 57–97, *p* = 0.0438). However, no significant correlation between time interval from CAR-T-cell therapy to the first vaccination and antibody outcome was observed. We also found no significant correlation between the age at time of vaccination and the antibody outcome. These results suggest that patients’ B-cells and CAR-T-cells are important prognostic parameters in order to find the optimal time for vaccination. An earlier study from Ram et al. produced similar results. They examined 66 hematopoietic cell transplantation patients and 14 patients with CAR-T-cell therapy and found that higher CD19+ cells were associated with positive humoral responses [13].

Patients with two doses of the vaccination before CAR-T-cell therapy (CAR-T after VAC) showed a slightly higher seropositivity rate after the second vaccine dose, as six patients (42.9%; 6/14) had positive humoral antibodies. However, it was only in one patient (12.5%; 1/8) that a negative result after the second vaccine could be turned into a positive result after the third dose, which took place as re-vaccination after CAR-T-cell therapy. Three patients (3/4) seroconverted after the fourth vaccination, while 1pt. (1/4) stayed negative after the third and fourth vaccinations. Therefore, our study suggests that patients with vaccination prior to CAR-T-cell therapy should receive two doses as re-vaccination after CAR-T-cell therapy. The reason for this is the higher benefit shown above in the administration of a fourth dose in comparison with only a third dose.

Given that patients were retrospectively divided into two groups (CAR-T before VAC/CAR-T after VAC), a comparison between these two groups is difficult as there were different amounts of patients in the two groups. Our study reflects a real-world scenario in which some of the patients had their CAR-T-cell therapy without the protection of a COVID-19 vaccination as vaccines were not available at the time. In these patients with CAR-T before VAC, only 23.3% (7/30) had a positive antibody test result after two vaccinations. In comparison, patients with CAR-T after VAC 42.9% (6/14) had a positive antibody test result after two vaccinations. Thus, we could assume that six patients with CAR-T after VAC had protection by a positive antibody test result as they went through the procedure of the CAR-T-cell therapy. We consider this as a benefit in vaccination before CAR-T-cell therapy.

Overall, 12 out of 46 patients reported a COVID-19 infection: 4 patients with positive antibody response and 8 patients with no detectable antibody response. Due to the limited patient count, no definite conclusions can be drawn as to the protection offered by different antibody levels.

Limitations to this study were the retrospective data collection and the limited number of patients, particularly at the fourth vaccination time point. Additionally, our study only examined the humoral antibody responses and not the cellular reactivity to COVID-19 vaccines. Currently, it also remains unclear which quantities of antibody titers are protective against severe courses of COVID-19 infection. It is also not conclusively clarified how long protection by high antibody titers lasts; more studies are needed to address this question. However, this study provides an important real-world analysis of COVID-19 vaccines in CAR-T-cell patients with two different vaccination procedures. This information shows the benefits of additional booster doses for these patients and can give guidance for the optimal time of vaccination. Our study is an important addition to the very limited data available in this group of patients. Further studies with more CAR-T-cell recipients, especially for third and fourth vaccination, are needed to conclusively confirm our results.

## 5. Conclusions

Our results indicate poor humoral antibody responses in patients with CAR-T-cell therapy and two doses of mRNA COVID-19 vaccines, which could be improved by administration of an additional third or fourth dose of the vaccine. Low B-cell counts and high CAR-T-cell numbers are associated with lacking antibody response and could therefore be an important parameter in choosing the optimal moment of vaccination. However, larger studies will be needed to ultimately clarify this question.

## Figures and Tables

**Table 1:** Clinical characteristics of the patients.

Parameter	Total (46 Patients)
Demographic characteristics	
Males: females (ratio)	28:18 (1.6)
Median age at the time of diagnosis (range)	58.5 (17–78)
Initial diagnosis	
Primary (de novo) DLBCL	24 (52%)
Secondary/transformed DLBCL	11 (24%)
Mantle cell lymphoma	7 (15%)
B-ALL	4 (9%)
Previous hematopoietic SCT before CAR-T-cell therapy	
Autologous SCT	21 (46%)
Allogeneic SCT	1 (2%)
Median age at the time of CAR-T-cell therapy (range)	64 (18–80)
CAR-T-cell-therapy type	
tisagenlecleucel	28 (61%)
axicabtagene ciloleucel	10 (22%)
brexucabtagen autoleucel	8 (17%)
Clinical course after CAR-T-cell therapy	
CRS	30 (65%)
CRES	12 (26%)
Tocilizumab therapy	21 (46%)
Steroid therapy	16 (35%)
Relapse after CAR-T-cell therapy	10 (22%)
Death	3 (7%)

DLBCL—diffuse large B-cell lymphoma; B-ALL—B-cell acute lymphoblastic leukemia; SCT—hematopoietic stem-cell transplantation; CAR-T—chimeric antigen receptor T-cell; CRS—Cytokine release syndrome; CRES—CAR-T-related Encephalopathy Syndrome.

**Table 2:** Parameters concerning vaccination.

Parameter	Total [46 Patients]	CAR-T before VAC [32 Patients]	CAR-T after VAC [14 Patients]
Vaccine			
Moderna (mRNA-1273)	33 (72%)	22 (69%)	11 (78.6%)
Pfizer/BioNTech (BNT162b2)	13 (28%)	10 (31%)	3 (21.4%)
Remission Status at time of 1st vaccination			
CR	21 (46%)	21 (65.6%)	0 (0%)
PR	5 (11%)	4 (12.5%)	1 (7.1%)
SD	0 (0%)	0 (0%)	0 (0%)
PD	13 (28%)	3 (9.4%)	10 (71.4%)
NE	7 (15%)	4 (12.5%)	3 (21.4%)
Median interval between CAR-T and respective VAC in days (range)			
1st VAC (range)		286 (23–975) [32 patients]	−76 (−278; −34)) [14 patients]
2nd VAC (range)		317 (51–1003) [32 patients]	−37 (−239; −8) [14 patients]
3rd VAC (range)		531 (262–882) [23 patients]	102 (54–248) [8 patients]
4th VAC (range)		757 (610–1026) [3 patients]	274 (179–314) [4 patients]
Median interval of antibody determination after respective VAC in days (range)			
2nd VAC (range)		57 (24–203) [30 patients]	40 (5–289) [14 patients]
3rd VAC (range)		59 (14–162) [23 patients]	40 (6–90) [8 patients]
4th VAC (range)		48 (47–59) [3 patients]	59 (23–64) [4 patients]

CR—complete remission; PR—partial remission; SD—stable disease; PD—progressive disease; NE—not evaluated; CAR-T—chimeric antigen receptor T-cell therapy; VAC—vaccination.

**Table 3:** Outcome of humoral antibody determinations after CAR-T-cell therapy and COVID-19 mRNA vaccine in patients with “CAR-T before VAC”.

CAR-T before VAC (32 Patients): Anti-Spike Protein IgG Antibodies (AU/mL)			
	after 2nd VAC (30 Patients)	after 3rd VAC (23 Patients)	after 4th VAC (3 Patients)
Results after 2 doses (no 3rd or 4th vaccination)			
Pat. 1–8	<12	x	x
Pat. 9	143	x	x
Results after 3 doses (no data on 2nd dose and no 4th vaccination)			
Pat 10–11	x	<12	x
Results after 2 and 3 doses (no 4th vaccination)			
Pat. 12–20	<12	<12	x
Pat. 21	<12	12.2	x
Pat. 22	<12	28.5	x
Pat. 23	<12	43.5	x
Pat. 24	<12	110	x
Pat. 25	<12	138	x
Pat. 26	17.2	20.2	x
Pat. 27	106	400	x
Pat. 28	129	<12	x
Pat. 29	152	>400	x
Results after 2, 3, and 4 doses			
Pat. 30	<12	<12	24.3
Pat. 31	26.3	<12	22.1
Pat. 32	190	>400	302

All results are in AU/mL; <12 AU/mL: negative anti-spike protein IgG antibody test result; >12 AU/mL: positive anti-spike protein IgG antibody test result; >100 AU/mL: clear positive result; VAC—vaccination; x—no data available.

**Table 4:** Outcome of humoral antibody determinations after CAR-T-cell therapy and the COVID-19 mRNA vaccine in patients with “CAR-T after VAC”.

CAR-T after VAC (14 Patients): Anti-Spike Protein IgG Antibodies (AU/mL)			
	after 2nd VAC (14 Patients)	after 3rd VAC (8 Patients)	after 4th VAC (4 Patients)
Results after 2 doses (no 3rd and 4th vacciantion)			
Pat. 1–3	<12	x	x
Pat. 4	80.2	x	x
Pat. 5	87	x	x
Pat. 6	>400	x	x
Results after 2 and 3 doses (no 4th vaccination)			
Pat. 7–8	<12	<12	x
Pat. 9	<12	47.6	x
Pat. 10	90.01	321	x
Results after 2, 3, and 4 doses			
Pat. 11	<12	<12	<12
Pat. 12	<12	<12	346
Pat. 13	25.5	42.9	30.4
Pat. 14	99.2	<12	400

All results are in AU/mL; <12 AU/mL: negative anti-spike protein IgG antibody test result; >12 AU/mL: positive anti-spike protein IgG antibody test result; >100 AU/mL: clear positive result; VAC—vaccination; x—no data available.

## Data Availability

No data supporting the reported results are deposited elsewhere. The data presented in this study are available on request from the corresponding author. The data are not publicly available due to institution-related patient identity restrictions.
